# Domestic Correspondence

**Published:** 1884-02

**Authors:** 


### Domestic Correspondence.

BOSTON LETTER.

BOSTON, December 20, 1883.

This city is amply supplied with hospitals and dispensaries, and it may not be amiss to give you an idea of the Boston Dispensary and its new building, it being one of the oldest in the country, having been organized in 1796. The apothecary of the Dispensary was directed " to procure a board to be placed at the front of his shop, with the words 'Boston Dispensary' painted thereon, with such other device as may be congenial to the institution, and corresponding with his ideas and fancy." He selected that of "The Good Samaritan," and since that time this symbol has been used. In a report of the institution for 1858 is found the following: "Tradition says that the artist, in his anxiety to illustrate the full import of the scene described in Luke x. 32, painted the Levite in the act of passing him by on the other side" When the work was finished, the managers detected in the Levite so striking a likeness to a certain clergyman of the town, that the figure was effaced.

The support of the institution is from the interest on its funds and annual subscriptions. Until within a few years all medicines were dispensed free of charge. With some misgivings the managers ordered a fee of 10 cents to be charged on all medicines dispensed, of course leaving it open to the physicians to remit this fee in cases of absolute want. The result has been watched with interest, and instead of a yearly deficit, a third source of income has been added. The original intent of the institution was to care for the sick at their own homes, and from 13,000 to

14,000 persons are annually provided for in this manner; but an average of 84 patients are treated each day at the "Central Office," in the various special departments, and this now offers a large field of observation, which is being utilized in clinical instruction by the "visiting staff." The work of the "Central Office" was temporarily removed to Washington street, while the new building was erected on the site of the old building, at the corner of Ash and South Bennet streets. The building is very nearly square, the dimensions being 65 feet 8 inches by 65 feet 4 inches, with an entrance porch on Ash street. The walls are brick, with sandstone trimmings, and with the unique arrangement of the chimneys, the gable corners, and slate-hipped roof, we have an effective design. The building contains a basement, first and second stories, and a commodious attic. The basement contains the furnaces, a separate boiler for supplying hot water to the various parts of the building, the coal bins, and a completely fitted laboratory for the compounding of pharmaceutical preparations. The first story has a hall 22 feet wide, which runs the entire length of the building, and is used as the waiting-room for patients, on one side of which are the dispensary proper, trustees' room, laboratories, and a consulting room, and on the other side is divided into five consulting rooms for the physicians in attendance. The second story contains a hall of similar dimensions to the one below, with an ample and attractive staircase leading thereto. On one side there is a large, unfinished apartment, an operating and lecture room; on the other side, five consultiug rooms for the physicians. The attic contains the janitor's apartments. The finish throughout is of ash, with floors of the best Southern pine. Soapstone sinks and hot and cold water are placed in every room. The building, in point of construction, is really second to none in Boston. Ash settees for patients, and a reproduction of the old Plymouth chair for the physicians, make the furnishings comfortable and ornamental.

The friends of the medical education of women have again made an effort in their behalf. When the "Medical School" moved into its new building, an appeal was made to "the faculty" to allow the old building to be used for a school for the medical education of women, a sort of "annex" to the present "medical school." "The faculty," however, declined, considering that the movement would not have a sufficiently strong financial backing. The Dental Department of Harvard University now occupies the old building.

Kairine, the new antipyretic, has been used at the City Hospital by several gentlemen. The drug reduces the temperature certainly and rapidly, but in two cases produced alarming symptoms of depression.

The various medical societies are fully engaged in their winter's work, and a medical man's evenings might be almost constantly occupied in attending the various meetings. The division of the "county society" into sections is working very advantageously, and some good work is the result. B.

---

### TREATMENT OF DENTAL CARIES WITH SOLIDIFIED CREOSOTE.

Creosote is a popular remedy used for the pains caused by caries of the teeth. As its excessive fluidity frequently occasions bad accidents in the mouth of the persons that use it, it can be solidified by the addition of a certain quantity of collodion, 10 grams to 13 grams of creosote. This mixture is more manageable than the simple creosote, and forms a varnish which closes the orifice of the tooth and prevents the air from influencing the dental nerve.—*Revue de Thèrapeutique.*

### CHLORIDE OF GOLD.

Mr. Martineau prescribes the following solution, in doses of two to three teaspoonfuls for the most obstinate cases of syphilis and tertiary ulcerations:

Chloride of gold........1 gram.
Chloride of sodium......1 gram.
Distil. water.......... 1 litre.

He reminds us that the chloride of gold and of sodium have been lauded for the sharp pains of ataxia, and that Bazin recommended them in scrofula.—*Le Praticien.*

